# Neuroprotective Potential of *Origanum majorana* L. Essential Oil Against Scopolamine-Induced Memory Deficits and Oxidative Stress in a Zebrafish Model

**DOI:** 10.3390/biom15010138

**Published:** 2025-01-16

**Authors:** Ion Brinza, Razvan Stefan Boiangiu, Iasmina Honceriu, Ahmed M. Abd-Alkhalek, Samir M. Osman, Omayma A. Eldahshan, Elena Todirascu-Ciornea, Gabriela Dumitru, Lucian Hritcu

**Affiliations:** 1 Department of Biology, Faculty of Biology, Alexandru Ioan Cuza University of Iasi, 700506 Iasi, Romania; ion.brinza@student.uaic.ro (I.B.); razvan.boiangiu@uaic.ro (R.S.B.); iasmina.honceriu@student.uaic.ro (I.H.); ciornea@uaic.ro (E.T.-C.); 2 Faculty of Medicine (for Boys), Al Azhar University, Cairo 11651, Egypt; ahmedahmed1.stu.1@azhar.edu.eg; 3 Department of Pharmacognosy, Faculty of Pharmacy, October 6 University, Giza 3232031, Giza Governorate, Egypt; phar-ma@o6u.edu.eg; 4 Department of Pharmacognosy, Faculty of Pharmacy, Ain Shams University, Abbassia, Cairo 11566, Egypt; oeldahshan@pharma.asu.edu.eg; 5 Center of Drug Discovery Research and Development, Ain Shams University, Cairo 11566, Egypt

**Keywords:** ADMET, PASS Online, drug likeness, pKCSM, *Origanum majorana* essential oil, volatile oil, memory, anxiety, oxidative stress, Alzheimer’s disease, dementia, scopolamine, *Danio rerio*

## Abstract

*Origanum majorana* L., also known as sweet marjoram, is a plant with multiple uses, both in the culinary field and traditional medicine, because of its major antioxidant, anti-inflammatory, antimicrobial, and digestive properties. In this research, we focused on the effects of *O. majorana* essential oil (OmEO, at concentrations of 25, 150, and 300 μL/L), evaluating chemical structure as well as its impact on cognitive performance and oxidative stress, in both naive zebrafish (*Danio rerio*), as well as in a scopolamine-induced amnesic model (SCOP, 100 μM). The fish behavior was analyzed in a novel tank-diving test (NTT), a Y-maze test, and a novel object recognition (NOR) test. We also investigated acetylcholinesterase (AChE) activity and the brain’s oxidative stress status. In parallel, we performed in silico predictions (research conducted using computational models) of the pharmacokinetic properties of the main compounds identified in OmEO, using platforms such as SwissADME, pKCSM, ADMETlab 2.0, and ProTox-II. The results revealed that the major compounds were trans-sabinene hydrate (36.11%), terpinen-4-ol (17.97%), linalyl acetate (9.18%), caryophyllene oxide (8.25%), and α-terpineol (6.17%). OmEO can enhance memory through AChE inhibition, reduce SCOP-induced anxiety by increasing the time spent in the top zone in the NTT, and significantly reduce oxidative stress markers. These findings underscore the potential of using *O. majorana* to improve memory impairment and reduce oxidative stress associated with cognitive disorders, including Alzheimer’s disease (AD).

## 1. Introduction

Alzheimer’s disease (AD) is characterized by distinct pathological hallmarks that significantly contribute to its neurodegenerative process. The primary features include the accumulation of amyloid-beta plaques [1] and neurofibrillary tangles [2,3], which are critical in understanding the disease’s progression and potential therapeutic targets. Although amyloid and tau pathologies are central to AD, emerging research suggests that other factors, such as neuroinflammation and vascular contributions, may also play significant roles in the disease’s complexity and progression [4].

Currently, AD has several treatment options, primarily aimed at managing symptoms rather than curing the disease. Cholinergic inhibitors, particularly cholinesterase inhibitors (ChEIs), like donepezil, rivastigmine, and galantamine, are Food and Drug Administration (FDA) approved and effective for mild to moderate AD, working by inhibiting the breakdown of acetylcholine [5,6], which is crucial for cognitive function. Memantine is used for moderate to severe AD, often in combination with ChEIs, to enhance mental function [7]. Although ChEIs can improve cognitive function and daily-living activities, their efficacy is often limited to slowing disease progression rather than reversing it. ChEIs primarily slow cognitive decline rather than halt or reverse it, which may lead to patient and caregiver frustration [8]. Individual responses to ChEIs can vary widely, complicating treatment plans [9]. Common side effects following ChEI medication include nausea, vomiting, and diarrhea, which can lead to dehydration and malnutrition [9,10]. Additionally, ChEIs can cause bradycardia and other cardiovascular complications because of increased cholinergic activity [9].

Essential oils are emerging as a promising alternative to conventional medications for AD, offering potential neuroprotective effects and symptom management [11].

*Origanum majorana* L. has shown potential therapeutic benefits for managing symptoms of AD because of its rich profile of bioactive compounds with neuroprotective properties. These compounds, including flavonoids, phenolic acids, and terpenoids [12], exhibit antioxidant, anti-inflammatory, and cholinergic activity modulations crucial in AD pathology. Research indicates that *O. majorana*’s phytochemicals, particularly rosmarinic acid and thymol, possess strong antioxidant and anti-inflammatory effects, which are critical in combating neuroinflammation and oxidative stress associated with AD [13,14].

*O. majorana* also contains a variety of primary and secondary phytoconstituents that contribute to its therapeutic properties. These include carvacrol, which exhibits antimicrobial and anti-inflammatory activities [15]; linalool, a terpene alcohol with calming and analgesic properties [15]; ursolic acid, a triterpenoid with anti-inflammatory and antioxidant effects [16]; and flavonoids such as apigenin, known for its anti-inflammatory and antioxidant effects [17], and luteolin, recognized for its antioxidant and anti-inflammatory activities [18]. Additionally, *O. majorana* contains a significant amount of tannins, which possess antioxidant and antimicrobial effects [15].

Additionally, *O. majorana* essential oil (OmEO) has been shown to enhance brain-derived neurotrophic factor (BDNF) expression, further supporting cognitive function improvement in AD models [14]. Histological analysis revealed reduced neurodegeneration in treated mice, indicating *O. majorana*’s protective role against brain damage [13]. Studies suggest that *O. majorana* extracts may inhibit acetylcholinesterase (AChE), an enzyme that degrades acetylcholine, thereby enhancing cholinergic signaling [19,20].

Scopolamine (SCOP), a muscarinic acetylcholine receptor antagonist, is widely used in experimental studies to induce cognitive and memory impairments that mimic aspects of AD [21]. Zebrafish (*Danio rerio*) has emerged as a powerful model organism for studying the effects of SCOP because of their genetic, biochemical, and behavioral similarities to humans [22].

This study aims to evaluate the potential of OmEO in ameliorating SCOP-induced memory impairment and mitigating brain oxidative stress, leveraging the zebrafish model as a versatile and effective platform for preclinical neuropharmacological research. This study highlights, for the first time, the detailed chemical composition of OmEO, including trans-sabinene hydrate, terpinen-4-ol, and linalyl acetate, and correlates these compounds with their pharmacological effects. Simultaneously, using in silico prediction methods, this study presents the pharmacokinetic and toxicological properties of the major compounds detected in OmEO. Additionally, through the combination of multiple behavioral tests (NTT, Y-maze, and NOR), this study provides a comprehensive evaluation of anxiety and memory, whereas most previous research has focused on a single type of behavioral test. Furthermore, it examines the effects of OmEO on AChE activity and oxidative stress, including markers such as superoxide dismutase (SOD), carbonylated proteins, and malondialdehyde (MDA), aspects that have been insufficiently explored in prior studies.

## 2. Materials and Methods

### 2.1. Plant Material and Essential Oil Preparation

*Origanum majorana* L. (the whole herb) was subjected to steam distillation in a Clevenger apparatus (AlBouroge, Cairo, Egypt) for 5 h. The produced oil has a pale yellow color and a herbaceous sweet odor. The oil was purchased in dark bottles from the Somitt Aromatic Company (Cairo, Egypt).

### 2.2. Gas Chromatography/Mass Spectrometry (GC/MS) Analyses

#### 2.2.1. GC/FID Analyses

GC/FID analyses were carried out on a Varian 3400 apparatus (Varian GmbH, Darmstadt, Germany) equipped with an FID detector and an Rtx-5MS fused–bonded silica column (30 m × 0.25 mm i.d.; film thickness: 0.25 µm; Ohio Valley, OH, USA); the operating conditions were as follows: The initial column temperature was kept at 45 °C for 2 min (isothermal) and then programmed to rise at a rate of 5 °C/min to 300 °C and held for 5 min. The detector and injector temperatures were 300 °C and 250 °C, respectively. The sample volume was 0.03 µL. The helium carrier gas flow rate was 2 mL/min. A Peak Simple 2000 chromatography data system (SRI Instruments, Torrance, CA, USA) was used for recording and integrating the chromatograms.

#### 2.2.2. GC/MS Analyses

These analyses were carried out on a Hewlett Packard gas chromatograph (GC HP 5890 II; Hewlett Packard GmbH, Bad Homburg, Germany) equipped with the same column and under the same conditions as those for GC/FID. The capillary column was directly coupled to a quadrupole mass spectrometer (SSQ 7000; Thermo-Finnigan, Bremen, Germany). The injector temperature was 250 °C. The helium carrier gas’s flow rate was 2 mL/min. All the mass spectra were recorded under the following analytical conditions: filament emission current, 60 mA; electron energy, 70 eV; ion source temperature, 200 °C; scan range from 40 to 400 Amu. The diluted samples (0.5% *v*/*v* n-hexane was used as the solvent) were injected in split mode (split ratio, 1:15). Compounds were identified by a comparison of their mass spectral data and retention indices with those in the Wiley Registry of Mass Spectral Data, 8th edition [23] and the NIST Mass Spectral Library (https://chemdata.nist.gov/, accessed on 1 May 2023). The identification was further confirmed by the calculation of the retention indices (RIs) relative to those of a homologous series of n-alkanes (C6–C22) under identical experimental conditions, as well as matching with those in the literature [24,25,26,27].

### 2.3. Estimated In Silico Pharmacokinetic Profiles of Compounds

For the computational analysis, we used the simplified molecular in-line insertion system (SMILES) for SCOP, galantamine (GAL), and the main compounds of OmEO (trans-sabinene hydrate, terpinen-4-ol, linalyl acetate, caryophyllene oxide, and α-terpineol), which were retrieved from the PubChem platform [28] in November 2023. Five free calculators were used, such as PASS Online [29], SwissADME [30], pKCSM [31], ADMETlab 2.0 [32], and ProTox-II [33]. To perform a comparison between predictions obtained from various websites and experimental data, it was essential to standardize the units of measurement (such as solubility, log S, and clearance). For the other parameters, such as absorbance and permeability, it was necessary to convert the data to binary categories (e.g., Yes/No). This was crucial to facilitate an evaluation of the predictions, as some websites used different classification levels.

The selected probabilities were 1. Caco2 permeability, 2. Intestinal absorption (human), 3. Skin permeability, 4. VDss (human), 5. BBB permeability, 6. CNS permeability, 7. CYP3A4 substrate, 8. CYP1A2 inhibitor, 9. Total clearance, 10. Renal OCT2 substrate, 11. Max. tolerated dose (human), 12. Oral rat acute toxicity (LD50), 13. Oral rat chronic toxicity (LOAEL), 14. Hepatotoxicity, 15. Skin sensitization, and 16. Respiratory toxicity.

### 2.4. Estimated Toxicity Pathways

To evaluate the toxicity pathways of SCOP, GAL, trans-sabinene hydrate, terpinen-4-ol, linalyl acetate, caryophyllene oxide, and α-terpineol, the ADMETlab 2.0 tools were used [34], as well as ProTox-II [35], which predictions are based on quantitative high-throughput screening (qHTS) assays and include a chemical library of over 10,000 compounds from the Tox21 data set. These screening tests mainly focus on nuclear receptors and how cells respond to stress [36].

### 2.5. Compound Prediction and Drug Analogy

To evaluate the structural criteria, for the optimal action in the body, of the studied compounds, the SwissADME online instrument was used [37], calculating the main drug design rules, such as Lipinski’s rule [38], Weber’s rule [39], Ghose’s rule [34], and Egan’s rule [35]. These computational methods are based on algorithms trained on large datasets and provide significant accuracy following cross-validation. Lipinski’s rule refers to the following criteria: (1) the molecular mass must be below 500 Da; (2) the maximum number of hydrogen bond donors is 10; (3) the maximum number of hydrogen bond acceptors is 5; (4) the octanol/water logarithm (Log P(o/w)) must be below 5 [38]. Weber’s rule refers to the number of rotational bonds (also called the rule complexity) in a molecule [39].

Ghose’s rule imposes the following limitations: (1) the molecular mass must be between 160 and 480 Da; (2) the octanol/water logarithm (Log P(o/w)) must be between 0.4 and 5.6; (3) the refraction index must be between 40 and 130; (4) the total number of atoms must be between 40 and 130, and this serves as a molecular descriptor [34].

Egan’s rule mainly focuses on liver toxicity and is based on the following parameters: (1) Liver toxicity index. This is calculated based on some chemical properties of the molecule and must be below a certain specified threshold; (2) The ratio of the water solubility to the octanol solubility (Log P(o/w)). This ratio must fall within a specific range to indicate a reduced likelihood of liver toxicity [35].

### 2.6. Animals and Study Design

This study aimed to highlight the neuropharmacological effects of OmEO on cognitive processes, anxiety-like behavior, and oxidative status in zebrafish. For this, 100 adult wild-type zebrafish (*Danio rerio*, Tubingen strain) were used, aged 5–7 months, which had short fins. The proportion of males to females was 1:1. The fish had an average body length of between 3 and 4 cm. All the fish in this study were purchased from the European Zebrafish Resource Center at the Institute of Toxicology and Genetics in Germany. Immediately after purchase, all the animals used in this study were quarantined in a 60 L tank and monitored for two weeks. After that, all the zebrafish were housed in groups of 10 individuals in a tank made of transparent glass, with a volume of 10 L of dechlorinated water, treated with Tetra AquaSafe (Tetra, Melle, Germany), which was replaced every two days. The water temperature was maintained at 28 ± 2 °C. The water quality was monitored twice a day (at 8 a.m. and 8 p.m.) and was maintained at the following parameters: pH between 7 and 7.5, dissolved oxygen in the range 8 ± 1 mg/L, conductivity between 1500 and 1600 µS/cm, ammonia and nitrite contents below 0.001 mg/L, and a standard lighting cycle of 14 h of light and 10 h of darkness. The animals were fed Norwin Norvitall flake (Norwin, Gadstrup, Denmark) three times a day (at 8 a.m., 2 p.m., and 8 p.m.), with equal amounts of food that could be consumed in 10 min by the fish. The air pump was turned off during feeding to facilitate the consumption of the food by the fish. Regarding ethics in animal research, all the animal manipulations were carried out under European Parliament Directives 2010/63/EU and research protocols approved by the Animal Research Ethics Committee at the Faculty of Biology, Alexandru Ioan Cuza University of Iasi, Romania (No. 1714/6 July 2023).

**Figure 1:**
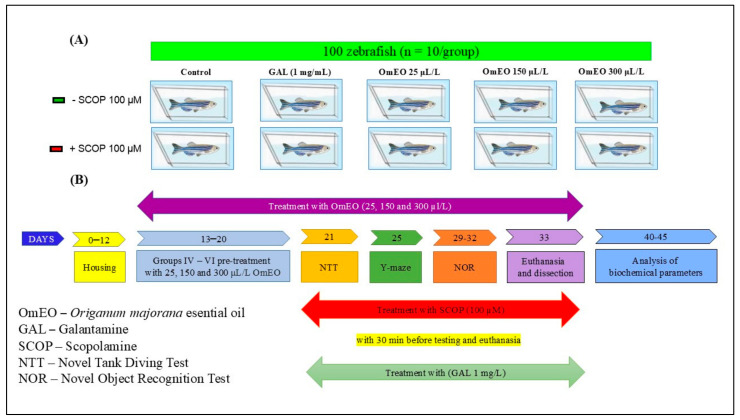
Schematic representation of the experimental design of this study. (**A**) Experimental groups; (**B**) behavioral and biochemical tests.

The animals were randomly divided into 10 experimental groups, as shown in Figure 1A: (I) the control group; (II) the group treated with galantamine (GAL, 1 mg/L), which served as a positive control group in behavioral and biochemical tests; (III, IV, and V) three treatment groups with OmEO at concentrations of 25, 150, and 300 μL/L; (VI) the group treated with scopolamine (SCOP, 100 μM); (VII) the group treated with SCOP (100 µM) + GAL (1 mg/L); (VIII, IX, and X) three groups treated with SCOP (100 µM) + OmEO at concentrations of 25, 150, and 300 µL/L. GAL (1 mg/L) was administered individually in a 500 mL Berzelius cylinder glass for 3 min before behavioral tests and euthanasia to both group (II) and group (VII) fish. OmEO was chronically administered, to fish in experimental groups III–V and VIII–X, after being dissolved in a 1% Tween 80 solution at concentrations of 25, 150, and 300 µL/L, with the tank’s water exchange. Treatment doses were selected based on previous reports [40], where it has been demonstrated that essential oils can be effective in modulating memory deficits in zebrafish, at low concentrations in the range of microliters per liter. A dementia-like state in the zebrafish was induced by SCOP (100 µM) treatment for 30 min before the NTT test and the euthanasia procedure. For the Y-maze and NOR tests, SCOP (100 µM) was administered 30 min after the training session. This was performed according to the methods already established and detailed in previous studies [40,41]. This concentration has been effective in inducing cognitive deficits and anxiety, as well as cholinergic impairments and oxidative imbalance.

In experimental group VII, the fish were subjected to an additional treatment with GAL (1 mg/L) for 3 min, after the administration of SCOP (100 µM), according to the illustration shown in Figure 1B. Also, we confirmed that *n* = 10 fish/group was appropriate using InVivoStat v4.10, an R-based statistical package v4.4.2 [42]. At a significance level of 0.05, the power to detect a 20% biologically relevant change is 98%.

To minimize bias, the number of fish per group was balanced (*n* = 10), and testing conditions were uniform. Additionally, the personnel who performed the evaluation of the biochemical and behavioral parameters were not informed about the treatments applied.

### 2.7. Behavioral Analysis

To evaluate the impact of OmEO on the behavior of the animals tested in this study, we monitored the activity of the zebrafish using a Logitech HD Webcam C922 Pro Stream digital camera (Logitech, Lausanne, Switzerland). The obtained videos were subsequently analyzed using ANY maze^®^ software 7.44, provided by Stoelting Co. in Wood Dale, IL, USA.

#### 2.7.1. Novel Tank-Diving Test

Zebrafish naturally exhibit anxiety when introduced to a new and unfamiliar environment [43]. To assess these behaviors, numerous tests have been developed, such as the novel tank-diving test (NTT), which capitalizes on the zebrafish’s natural diving response, is well adapted to assess anxiety levels, and presents, at the same time, a solid face-validity [44,45,46]. The NTT is also construct-validated, as both anxiolytic and anxiogenic drugs have been shown to impact the intensity of fish responses in their tasks [47]. This underscores the value of using NTT in anxiety-related studies in zebrafish [48].

**Figure 2:**
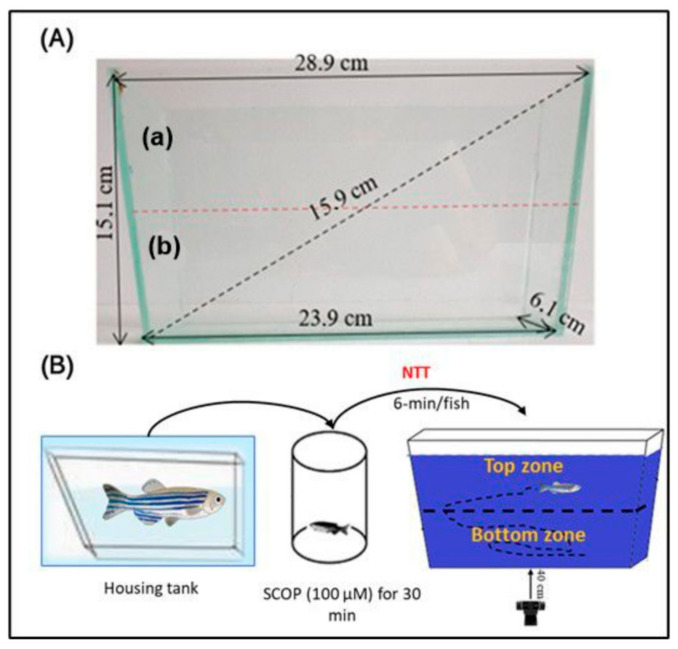
Novel tank-diving test (NTT). (**A**) The tank used to evaluate the behavior of the zebrafish in the NTT test, as well as its dimensions: (a) the top zone of the tank and (b) the bottom zone of the tank. (**B**) Experimental setup of the test.

To evaluate the impacts of OmEO on the anxiety states of both the control zebrafish and those treated with SCOP (100 µM) in the NTT, as well as those undergoing acute treatment with SCOP (100 µM) in the NTT, the protocol established by Cachat et al. [49] was used. Thus, a tank with trapezoidal walls was used, made of glass with perfect transparency (Figure 2A). The dimensions of this tank were as follows: a height of 15.1 cm, a base length of 23.9 cm, a length at the top of 28.9 cm, and a width of 6.1 cm. The aquarium was practically divided into two equal horizontal sections: one upper and the other lower (Figure 2A).

During the test, the tank was filled with 1.5 L of water taken from the fish’s housing tank and placed on a flat white surface. Each fish specimen was tested individually, once, for 6 min (Figure 2B). Within this period, anxiety-like behavior was measured as functions of the latency period (s), time (s), and distance spent in the top zone of the tank (m). The formula used to calculate the latency to enter the top zone (s) is (Time of the first entry in the top zone) − (Start time of the test). Also, in the NTT, we assessed locomotion-related phenotypes of the zebrafish by recording the following parameters: freezing duration (s) and mean swimming speed of the fish (m/s). Therefore, these measurements allowed us to investigate both the anxiety-like behaviors and the locomotor responses of the fish in the novel environment.

#### 2.7.2. Y-Maze Test

The zebrafish is used as a pharmacological model for studying learning and memory performances, and assessing these aspects involves monitoring several types of behaviors, including locomotion (how the zebrafish moves and explores its environment), anxiety-like emotion (the fish’s reactions to stressful or threatening situations), and cognition (assessment of cognitive abilities, such as learning, object recognition, and spatial memory) [50]. As a model organism, the zebrafish does not allow for the assessment of all forms of memory. Like rodent models, zebrafish are mainly used to study three common types of memory: spatial memory, recognition memory, and associative memory. To assess memory, a delay period is required between the training phase (familiarization) and the actual testing phase [51]. Using the Y-maze has several significant advantages. First, it is a simple and effective method of memory assessment, and training sessions can be carried out quickly. This allows for specific memory training without involving complex conditioned learning. In addition, the Y-maze minimizes potential factors that could influence animal performance, such as emotional and motivational states. Through this approach, a clearer and more focused assessment of the memory and behavioral responses of animals can be obtained in a controlled and standardized environment [52].

**Figure 3:**
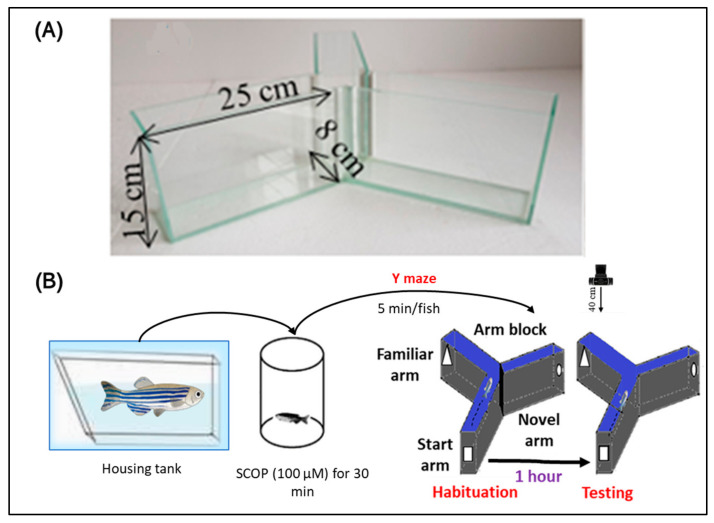
Y-maze. (**A**) The tank used to evaluate the behavior of the zebrafish in the Y-maze test, as well as its dimensions; (**B**) experimental setup of the test.

To assess the effects of OmEO on spatial memory and novelty responses in zebrafish, we used the Y-maze test according to the protocol established by Cognato et al. [53]. The tank used for the Y-maze was made of transparent glass in a Y-shape. The tank was made up of three equal arms, each 25 cm long, 8 cm wide, and 15 cm high, forming an angle of 120° (Figure 3A). To facilitate video analysis, the floor of the tank was covered with white plastic material, while the remaining areas were covered with black plastic, with visual cues attached to the interior in the form of geometric shapes, such as squares, circles, and triangles (Figure 3B). The first (training) session consisted of a 5 min period in which the fish were allowed to explore only two of the three arms of the maze, namely, the start arm and the familiar arm, while the third arm, representing the novel arm, was blocked in this phase (Figure 3B). In the Y-maze test, the center of the maze was considered as a preselection area and was not included in the analysis and interpretation of the results. The test session was performed one hour after the training session (Figure 3B). During the test session, the fish were again placed in the same start arm, but with free access to all three arms, for 5 min. The ability of the fish to respond to novelty was recorded using a camera mounted above the tank, on the ceiling. The recordings were subsequently examined to determine the spontaneous alternation (%), turn angle (°), and the percentage of the time spent by the fish in the novel arm relative to the start arm and the familiar arm (%). The formula for calculating the percentage of the spontaneous alternation is (Number of alternations/Total possible alternations) × 100. To calculate the percentage of the time spent by the fish in the novel arm relative to the start arm and the familiar arm, the following formula was used: (Time spent in the novel arm/Total time spent in all arms) × 100.

#### 2.7.3. The Novel Object Recognition Test

The novel object recognition test (NOR) has become a fundamental tool in the research of the neurobiology of memory. These studies are used to investigate a wide range of memory processes, such as the acquisition, consolidation (and reconsolidation), and re-acquisition of information, while allowing for detailed disclosure of the brain mechanisms involved in these processes [54].

**Figure 4:**
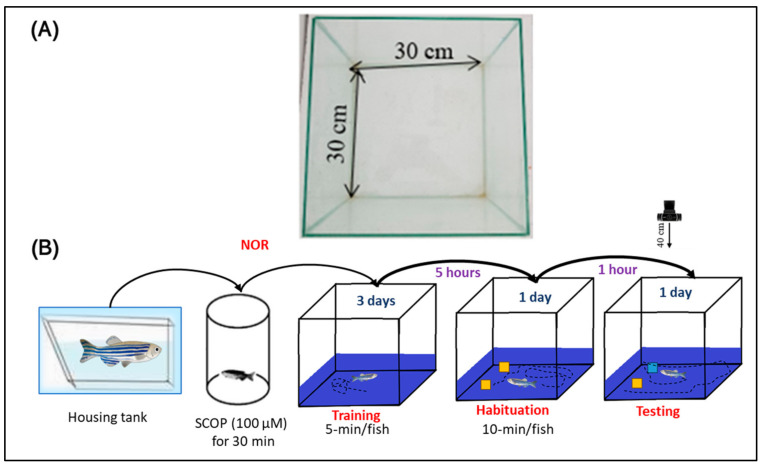
The novel object recognition test (NOR). (**A**) The tank used to evaluate the behavior of the zebrafish in the NOR test, as well as its dimensions; (**B**) experimental setup of the test.

To determine whether OmEO has the potential to exert effects on the zebrafish’s reinstatement memory, the NOR test was used according to the protocol established by Stefanello et al. [55]. For this test, we used a cube-shaped glass tank with a width and a height of 30 cm (Figure 4A). The outer walls of the tank were covered with black fabric, both to reduce stress on the fish during testing and to minimize the experimental error. The tank was placed on a flat surface and filled with water from the fish housing tank, with the water level reaching 5 cm from the upper edge of the test tank. This water level was chosen to allow the fish to swim horizontally, thereby reducing their vertical activity during the test. The test was conducted over four days (Figure 4B). During the first three days, each fish was allowed to habituate to the test tank for 5 min twice a day. The interval between habituation periods was 5 h. The training session was conducted 12 h after the last habituation session and involved the exploration of two identical objects (yellow cubes of 2.5 centimeters per side) for 10 min. The cubes were placed in two corners of the tank and oriented parallel with 10 cm between them. After the training session, there was a one-hour retention period. During this, one of the yellow cubes was replaced by a white one. Thus, the yellow cube was considered as familiar (F), and the blue cube was considered as new (N). In the test session, each fish was allowed to explore the two cubes for 10 min. The fish’s recognition memory was assessed as a percentage of the fish’s preference. The fish’s preference (%) for one of the two cubes (calculated as the difference between the time spent exploring the novel cube vs. the familiar cube) was determined using the following formula: (Time spent exploring the novel cube)/(Time spent exploring the familiar cube + Time spent exploring the new cube) × 100.

### 2.8. Preparation of Homogenates and Analysis of Biochemical Parameters

Directly following the end of the last behavioral test, the study’s animals were individually transferred to glass containers filled with ice-cold water (at a temperature range of 2–4 °C) for a duration of 10 min. Subsequently, the animals were promptly and humanely euthanized through decapitation, following established procedures, as previously outlined [40]. The entire brains were meticulously removed, weighed, and transferred to 0.5 mL tubes. These samples were then stored at a low temperature of – 20 °C until they were to be used in subsequent procedures.

On the following day, the brains of the fish from the same groups of animals were weighed individually (within a range of approximately 3–6 mg each) and then homogenized in a phosphate extraction buffer (0.1 M potassium phosphate buffer, pH 7.4, in which KCl was added at a concentration of 1.15%) at a ratio of 1 to 10. This homogenization process was carried out using a ball mill (Mikro-Dismembrator U; Sartorius, NY, USA). After homogenization, the resulting suspension was centrifuged for 15 min at a speed of 14,000 rpm, and the supernatant was later used to determine biochemical parameters.

### 2.9. Determination of Acetylcholinesterase Activity

To assess AChE activity in the zebrafish brain homogenates, we used the photometric method detailed in Ellman’s paper [56]. An acetylthiocholine iodide (ATCh) mixture (purchased from Sigma, St. Louis, MO, USA) and the dithiobisnitrobenzoic acid (DTNB) reagent (purchased from Sigma, St. Louis, MO, USA) were combined in a phosphate buffer (pH 7.4). The ATCh iodide was subjected to hydrolysis, generating thiocholine and acetate ions. Afterward, the spectrophotometric method was used to measure the enzymatic activity at a wavelength of 412 nm by monitoring the rate of development of a yellow color produced following the reaction between thiocholine and DTNB. The enzymatic activity was expressed in nanomoles of ATCh per minute per milligram of protein. The protein content of the brain samples was determined using the Bradford method [57].

### 2.10. Determination of Superoxide Dismutase Activity

In this study, we assessed the superoxide dismutase (SOD)-specific activity using a protocol adapted from Winterbourn et al. [58]. The ability of this enzyme to inhibit the reduction of nitroblue tetrazolium (NBT) by superoxide free-radicals, generated in the reaction medium by the photoreduction of riboflavin, was measured. The samples contained 0.067 M potassium phosphate, the enzyme extract, 0.1 M EDTA, 0.12 mM riboflavin, and 1.5 mM NBT, and the absorbance was read at 560 nm. The specific activity of the SOD was expressed in enzyme units per milligram of protein, as determined using the Bradford method [57]. Thus, we efficiently assessed the enzymatic activity in a tailored and standardized way.

### 2.11. Determination of Catalase Activity

To assess the catalase (CAT) activity, we applied a simple colorimetric method originally described by Sinha [59]. First, 125 µL of the enzyme homogenate and 125 µL of a 0.16 M H_2_O_2_ substrate solution were pipetted into reaction cells. After 3 min, the reaction was stopped by adding 500 µL of a potassium dichromate–glacial acetic acid solution, and the tubes were incubated at 95 °C. After 10 min, the tubes were centrifuged at 14,000 rpm for 5 min, and the absorbance of the supernatant was read at 570 nm. The enzymatic activity was expressed in picomoles of H_2_O_2_ consumed per minute per milligram of protein. The amount of protein was determined using the Bradford method [57].

### 2.12. Determination of Glutathione Peroxidase Activity

In this study, the glutathione peroxidase (GPX)-specific activity was determined according to the procedure detailed by Fukuzawa and Tokumura [60]. This method is based on the ability of GPX to catalyze the decomposition of H_2_O_2_, using GSH as a reducing agent. This reaction generates products such as oxidized glutathione (GSSG) and water. The procedure involved the addition of specific volumes of the enzyme extract, sodium phosphate buffer, EDTA solution, and NaN_3_ solution to individual 1.5 mL tubes, as previously reported [40]. After a 10 min incubation at 37 °C, each tube was supplemented with GSH and H_2_O_2_ solutions, followed by another incubation step at the same temperature for 10 min. The reaction was then stopped with metaphosphoric acid, and centrifugation steps followed to obtain the supernatant. To assess the enzymatic activity, the supernatant was transferred to new tubes, to which solutions of disodium phosphate and DTNB were added. After a precise interval of 10 min, the absorbance was measured at 412 nm. This was compared to that of a reference mixture that included distilled water, Na_2_HPO_2_ solution, and DTNB solution. The result was the calculation of the specific activity of GPX, expressed in enzyme units per milligram of protein; the amount of protein was determined using the Bradford method [57].

### 2.13. Determination of the Reduced Glutathione Content

In this study, we adopted the method detailed by Salbitani et al. [61] to determine the content of the reduced glutathione (GSH). For this, 70 µL of distilled water, 1100 µL of 0.3 M Na_2_HPO_4_ solution, 200 µL of the homogenate, and 130 µL of 0.04% DTNB solution were added to the microtubes. After mixing the reagents in the tubes, the GSH content was measured spectrophotometrically at 412 nm against the control solution (which did not contain the homogenate). Finally, the amount of GSH was related to the protein concentration and was expressed as micrograms of GSH per milligram of protein. The amount of protein was determined using the Bradford method [57].

### 2.14. Determination of the Carbonylated Protein Level

The degree of oxidation of the brain proteins was assessed by measuring the content of the proteins’ carbonyl groups, using a method originally described by Oliver et al. [62]. After obtaining the supernatant fraction, it was divided into two equal parts, each containing approximately 2 mg of protein. Both portions were subsequently subjected to precipitation using 10% trichloroacetic acid (TCA), until the final concentration was reached. Another sample was treated with 2 N hydrochloric acid, while a third sample was treated with a 0.2% dinitrophenylhydrazine (DNPH) solution in 2 N hydrochloric acid at an equivalent volume. Both samples were then incubated at 25 °C and shaken at 5 min intervals. The results obtained were expressed as nanomoles of carbonyl groups per milligram of protein. This is a measure of the level of protein oxidation in the brain sample analyzed.

### 2.15. Determination of the Malondialdehyde Level

The assessment of the level of lipid peroxidation (the MDA level) in the zebrafish’s brain was carried out according to the protocol detailed by Ohkawa et al. [63]. This method is based on the interaction of lipid peroxides in animal tissues with thiobarbituric acid, generating a pink color. The intensity of this color was measured at 532 nm. This procedure provides an accurate assessment of the degree of lipid peroxidation in the analyzed brain samples, thus allowing the investigation of the level of oxidative stress in zebrafish brain tissue.

### 2.16. Statistical Analysis

The results were presented as means ± standard errors of the mean (SEMs). Differences between group means were analyzed using two-way analysis of variance (ANOVA) followed by Tukey’s post hoc test, considering the treatment factor. Statistical significance was set at the *p* < 0.05 level. For statistical analyses, we used GraphPad Prism 9.4 software (GraphPad Software, Inc., San Diego, CA, USA). We also evaluated correlations between behavioral scores and both enzymatic activities and lipid peroxidation levels, using the Pearson correlation coefficient (r).

## 3. Results

### 3.1. Chemical Composition of the Origanum Majorana Essential Oil

A total of 17 components were identified by GC/MS analysis, representing 93.52% of the OmEO, as depicted in Table 1. The major compounds were trans-sabinene hydrate (36.11%), terpinen-4-ol (17.97%), linalyl acetate (9.18%), caryophyllene oxide (8.25%), and α-terpineol (6.17%).

### 3.2. Pharmacokinetic Profiles of Compounds

**Figure 5:**
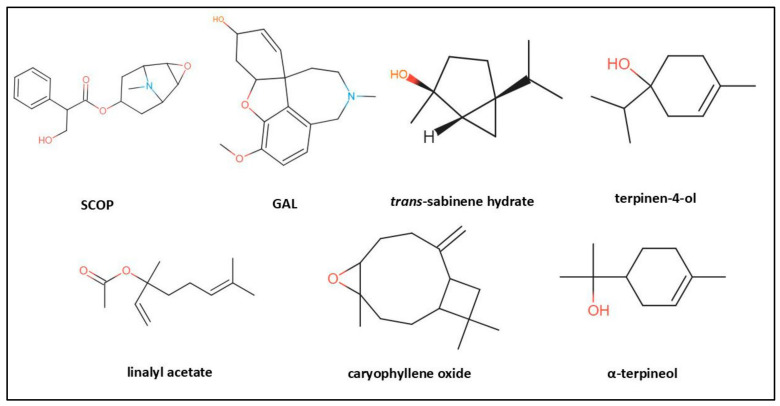
The chemical structures of scopolamine (SCOP), galantamine (GAL), and the main compounds of OmEO (trans-sabinene hydrate, terpinen-4-ol, linalyl acetate, caryophyllene oxide, and α-terpineol).

The ADMET properties, such as absorption, distribution, metabolism, excretion, and toxicity, of a chemical compound are crucial aspects in evaluating its drug potential. Although there are experimental methods for evaluating these properties, computational approaches are becoming increasingly popular because of their efficiency in terms of cost and time required for analysis. Some of these ADMET properties are particularly relevant in the development of treatments for diseases, such as AD, and for compounds with neuroprotective potential [64]. There is no ideal pharmacokinetic behavior that applies to all drug candidates, as it depends on the specific requirements of the target treatment. This variety of pharmacokinetic requirements is because of the diversity of treatments. To achieve an optimal therapeutic effect, drugs must have appropriate pharmacokinetic characteristics for their specific target. In this context, the pharmacological properties of the drug are essential to achieve the desired biological effect while minimizing the associated adverse effects [65]. To predict the properties of SCOP, GAL, and the main compounds of OmEO (trans-sabinene hydrate, terpinen-4-ol, linalyl acetate, caryophyllene oxide, and α-terpineol) (Figure 5), five different platforms were used, namely, PASS Online [29], SwissADME [30], pKCSM [31], ADMETlab 2.0 [32], and ProTox-II [33].

The interpretation of ADMET data expressed in Numeric (log Papp in 10^−6^ cm/s) indicates that substances with values higher than the threshold of 0.9 have an efficient Caco2 permeability, and those with values below 0.9 present from low to no Caco2 permeability. In terms of intestinal absorption, substances with an absorption of less than 30% are poorly absorbed. Also, for a molecule to have skin permeability, it must have a log Kp value of >−2.5. Our predictive pharmacogenomic profile results (shown in Table 2) indicate that GAL, trans-sabinene hydrate, terpinen-4-ol, linalyl acetate, caryophyllene oxide, and α-terpineol all show high absorption rates, as assessed by Caco2 permeability, intestinal absorption (human), and skin permeability (except for GAL, with a log Kp value of >−3.75). Regarding SCOP, our prediction data show that it has low Caco2 permeability (0.059) and low skin permeability (−4.097) but a high intestinal absorption rate (72.626).

To predict the distributions of substances in the body after administration, we calculated volume of distribution (VDss), fraction unbound (human), BBB permeability, and CNS permeability. Typically, parameters that have a significant impact on the blood concentration profile of a drug during intravenous administration include VDss, which measures how the drug spreads in the human body [66]. It is considered that VDss is reduced when it registers values below 0.71 L/kg (log VDss < −0.15), and it is considered as increased when it registers values above 2.81 L/kg (log VDss > 0.45). Thus, terpinen-4-ol and linalyl acetate present average VDss values (log VDss 0.21 and 0.069), and the other compounds present high VDss values (Table 2). A drug that is not bound in plasma has the potential to exert its pharmacological activity by interacting with various targets, such as proteins, enzymes, receptors, and channels. In the development of a pharmacokinetic model, the unbound fraction in plasma (“f u, p” value) of the drug represents a key factor in determining the drug’s efficacy. The “f u, p” value has also been found to influence several other aspects of drug efficacy and the occurrence of side effects, ranging from renal glomerular filtration to total clearance and hepatic metabolism [67]. Our prediction shows that the smallest fraction is caryophyllene oxide (0.327 Fu) and that the largest is α-terpineol (0.565 Fu).

The blood–brain barrier (BBB) is a selective and semipermeable barrier that maintains internal balance in the central nervous system (CNS). Assessing the permeability of compounds across the BBB is a crucial aspect of the development of CNS-targeted drugs, but it is a difficult problem to summarize in simple terms. Molecules with a logBB value of <−1 are very poorly distributed in the brain, while those with a logBB value of >3 can easily cross the BBB [68]. Our prediction data showed that all the OmEO compounds present the potential to cross the BBB, while SCOP and GAL are less distributed in the brain.

Although the parent compounds exhibit low permeability, their metabolites may have the potential to cross the BBB. Some metabolites could possess increased lipophilicity or may serve as substrates for active transport systems, thereby exerting effects at the level of the CNS [69]. However, cognitive improvements may be mediated through indirect pathways rather than direct neuronal modulation. For example, systemic reduction of oxidative stress or inflammation could enhance neuronal health and cognitive function [70]. Changes in systemic biochemical markers (e.g., oxidative stress markers and inflammatory cytokines) may reflect peripheral actions that indirectly influence CNS homeostasis [71]. Additionally, certain bioactive compounds may impact the synthesis, release, or degradation of critical neurotransmitters involved in cognition, such as acetylcholine, dopamine, serotonin, and glutamate, or modulate the activities of NMDA and AMPA receptors [72,73]. Another mechanism by which biochemical agents may support cognitive function is through their potential to enhance vasodilation and reduce platelet aggregation, thereby increasing the flow of oxygen and nutrients to the brain [74,75].

CNS permeability can be predicted by calculating the product of the BBB permeability and the specific surface area, expressed as logPS. In other words, to determine whether a compound can enter the CNS, a value called −2logPS is used. If the −2logPS value for a given compound exceeds a certain threshold or limit, typically −2logPS > −2, then that compound is considered to have the potential to successfully cross the BBB and enter the CNS. Conversely, compounds with −3logPS values are considered as unable to penetrate the CNS [76]. Thus, −2logPS serves as an indicator of the CNS permeability of a compound. By and large, as seen in Table 2, except for SCOP, which presents a low permeability for the CNS, GAL and the five compounds within OmEO present average or good permeabilities of the CNS.

CYP3A4 is considered as the most important detoxifying P450 enzyme because of its ability to facilitate the metabolism of a wide variety of xenobiotics with different structures to facilitate their excretion [77]. This includes over 50% of all the clinically relevant drugs currently in use, including codeine, diazepam, and chloroquine. CYP3A4 is also involved in the metabolism of some steroids and carcinogens. Some drugs can be activated by P450, and others can be deactivated [78]. As can be seen in Table 2, SCOP and GAL interact with the CYP3A4 substrate, while trans-sabinene hydrate, terpinen-4-ol, linalyl acetate, caryophyllene oxide, and α-terpineol do not interact with the CYP3A4 substrate. CYP1A2 is also an enzyme in the cytochrome P450 family and plays an important role in the metabolism of drugs and other chemicals in the body. Xenobiotic substrates for CYP1A2 enzymes include a wide range of chemical compounds, including caffeine, aflatoxin B1, and acetaminophen. These compounds are metabolized by CYP1A2 in the biotransformation process, which may affect how these xenobiotics affect the body [79]. Among the compounds tested by us, only caryophyllene oxide can interact with the CYP1A2 substrate.

Clearance is a metric used to assess how quickly a drug or substance is removed from the bloodstream. It is calculated by relating the elimination rate (expressed in milligrams per minute) to the concentration of the substance in the blood (expressed in milligrams per /milliliter). The total clearance is the sum of all the clearances from different organs and tissues that contribute to the elimination of the drug from the body [80]. ADMET and PkCSM predictions suggest that clearance has the highest values for linalyl acetate (1.627), followed by terpinen-4-ol (1.269), α-terpineol (1.219), SCOP (1.096), trans-sabinene hydrate (1.011), GAL (0.991), and caryophyllene oxide (0.905), all these values being expressed in logarithmic units (logarithm of milliliters per minute per kilogram), according to Table 2. Organic cation transporter 2 (OCT2) is a transporter that deals with the absorption of cation organics and is encoded by the *SLC22A2* gene. This OCT2 transporter is expressed in the kidney, specifically on the basolateral membrane of epithelial cells in the proximal tubules. Because its primary function is to transport substrates from the blood to the kidneys, OCT2 has a significant role in the process of the renal elimination of these compounds from the body [81]. Our predictions indicated that among the analyzed compounds, only GAL can interact with the OCT2 substrate.

Evaluations of drug toxicity and safety are an essential aspect of the drug discovery and development processes. Drug toxicities in organs, such as the liver, heart, kidneys, and brain, currently account for over 70% of the reasons drugs are withdrawn from the market [82].

Substances with a log milligram per kilogram per day value of less than or equal to 0.477 are considered to have low tolerance in humans, while those exceeding this value are considered to have high tolerance. According to ADMET and PkCSM, SCOP (−0.319), GAL (−0.423), and caryophyllene oxide (0.148) show low tolerances, and trans-sabinene hydrate (0.637), terpinen-4-ol (0.857), linalyl acetate (0.547), and α-terpineol (0.886) have high tolerances. Among the compounds studied, caryophyllene oxide showed the highest acute oral toxicity in rats (an LD50 value of 1,548 mol/kg), while GAL had the lowest acute oral toxicity (2728 mol/kg) according to Table 2. Concerning chronic oral toxicity in rats (LOAEL), it was found that in the case of the chronic administration of these three compounds, the lowest tolerated concentration was for SCOP (0.736 mg/kg_b.w./day), while the highest was for linalyl acetate (2256 mg/kg_b.w./day) according to Table 2.

Also, our predictions indicate that among the analyzed compounds, only GAL may have hepatotoxicity. Regarding skin sensitization, SCOP and GAL do not show sensitization; instead, the five compounds in OmEO can cause an allergic response in susceptible individuals. Substances are carcinogenic if after being inhaled, ingested, applied to the skin, or injected, they can induce the formation of malignant tumors, increase their incidence or malignancy, or shorten the time required for tumor development [83]. As can be seen in Table 2, α-terpineol and terpinen-4-ol may be carcinogenic. Simultaneously, SCOP, GAL, and linalyl acetate can induce toxic responses in the respiratory system.

### 3.3. Effects of OmEO Compounds on the Toxicity Pathway

In the field of toxicological research, machine-learning approaches and chemical structure descriptors have been used to predict toxicity-related biological activities. In this study, the toxicity-related proteomic features of the predominant compounds in OmEO were analyzed based on machine-learning models to predict the most significant features of the proteins involved in the toxicities of compounds in the Tox21 and ADMET2.0 datasets. Tox21 is a part of the therapeutic discovery platform CANDO (computational analysis of novel drug opportunities). Tox21 comprises over 10,000 compounds and provides diverse information, including twelve in vitro assays, seven related to nuclear receptor (NR) signaling, and five from the stress response (SR) pathway, but is characterized by significant imbalance among compound classes [84]. Herein, we used Tox21 to predict the activities of compounds (either toxic or nontoxic), thus identifying which of the chemical compounds in our study can affect the functions of six nuclear receptors (AR, AR-LBD, ER, ER-LBD, AhR, and PPAR-gamma) and five stress response pathways (ARE, ATAD5, HSE, MMP, and p53) in the human body. The data obtained after running ADMETlab 2.0 and ProTox-II are presented in Table 3.

### 3.4. The Similarities Between the Pharmacokinetic Properties of the Compounds

For medicinal chemical properties, we selected five complementary drug-likeness rules, including Lipinski’s rule [38], Weber’s rule [39], Ghose’s rule [34], and Egan’s rule [35], and the bioavailability score was also calculated. These rules have evolved in the long-term drug development processes of world-renowned pharmaceutical companies [85].

For the mentioned compounds, calculations of physicochemical properties, such as the molecular weight, hydrophobicity, numbers of hydrogen bond donor and acceptor atoms, number of rotatable bonds, polar molecular surface area (Table 4), and others, were performed. When excesses in these properties are identified for chemical compounds, the compounds are considered not to meet the same criteria as the drug. This analysis contributes to the evaluation of the drug potentials of these compounds [86].

The calculated drug-likeness and medicinal chemistry properties of the selected compounds are shown in Table 5. According to the results obtained, the SCOP and GAL compounds complied with all five filters (Lipinski, Ghose, Veber, Egan, and Muegge) used, without any violations. In contrast, the other compounds predominantly found in OmEO (trans-sabinene hydrate, terpinen-4-ol, linalyl acetate, caryophyllene oxide, and α-terpineol) registered at least a violation of one of these drug-like filters. The Abbot bioavailability score calculated for all the compounds placed them in the 55% probability class, as can be seen in Table 5.

### 3.5. Effects of OmEO on Anxiety-like Behavior in the Novel Tank-Diving Test

Anxiety, a feature present in many affective disorders, represents an important research topic for the development of new drugs or the reevaluation of existing ones [87]. To investigate this issue, zebrafish have been widely used in neuroscientific research on affective disorders, with a particular focus on the study of anxiety [46]. The novel tank-diving test (NTT) is a valued approach used by numerous research teams and groups to analyze swimming behavior in the context of anxious or stressful states [43]. The behavior of adult zebrafish in the NTT implies that they initially sink and stay at the bottom of the tank and go through a gradual adaptation to the rest of the space. This specific behavior was associated with a preemptive antipredator response, followed by a reduction in anxiety levels. This interpretation was based on the observation that under laboratory conditions, acclimated zebrafish spend most of their time at the surface of the tank during the day [48,88].

**Figure 6:**
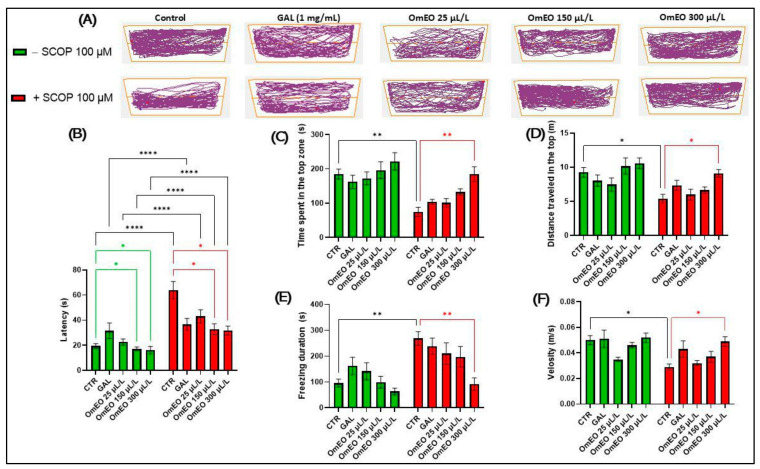
The effects of administering a treatment of *Origanum majorana* essential oil (OmEO), at concentrations of 25, 150, and 300 μL/L, both on native zebrafish and those treated with scopolamine (SCOP, 100 µM) in the NTT test. Galantamine (GAL, 1 mg/L) was used as a positive control (**A**). Graphical representation of zebrafish swimming in the NTT test. (**B**) The latency period required for the fish to start exploring the top zone of the tank (s). (**C**) The time spent by the fish in the top zone of the tank. (**D**) The distance traveled by the fish in the top zone of the tank. (**E**) The freezing duration. (**F**) The velocity of the zebrafish in the novel tank-diving test (NTT). Values are expressed as means ± S.E.M. (*n* = 10 animals per group). For Tukey’s post hoc analyses, * *p* < 0.05, ** *p* < 0.01, and **** *p* < 0.0001.

In the NTT test, distinct patterns of locomotor behavior are observed during the full 6 min session (Figure 6A). The control group displays typical zebrafish behavior in the NTT. In contrast, the group treated with SCOP (100 µM) reveals an increased preference for the bottom zone. Regarding the patterns of locomotor behavior in zebrafish treated with different concentrations of OmEO (25, 150, and 300 μL/L), we observe that they show locomotor behavior like that of the control group. A similar behavior was also recorded in OmEO-treated fish, at the mentioned concentrations, and subsequently subjected to SCOP (100 μM) treatment. Likewise, the group treated with GAL (1 mg/L) and the one treated with SCOP (100 µM) + GAL (1 mg/L) presented locomotor activities like that of the fish in the control group. Tukey’s post hoc analysis showed that treatment with SCOP (100 μM) increased the latency period (*p* < 0.0001) (Figure 6B), decreased the latency time (*p* < 0.01) and the distance (*p* < 0.05) spent by the fish in the top zone (Figure 6C,D), increased the periods of freezing (*p* < 0.01) (Figure 6E), and decreased the mean swimming speed (*p* < 0.05) (Figure 6F). All these behavioral changes indicate the induction by SCOP of an anxiogenic and hypolocomotor response.

The SCOP effects we observed in this study are contrary to those previously published by Hamilton et al. [89], where the authors demonstrated that SCOP produces anxiolytic effects, describing a biphasic relationship, which reached the maximum effect at a concentration of 800 µM. Also, in their study, SCOP (at a concentration of 800 µM) showed an anxiolytic effect in a group behavioral test, significantly reducing the animals’ tendency to escape. However, the effects of using SCOP (100 µM) may be because of its lower concentration and its acute administration. In this way, de Abreau et al. [90] described that acute administration of SCOP in humans can cause confusion, anxiety, fear, agitation, and irritability, possibly because of the superimposition or inclusion of its well-known excitatory action, which can lead to delirious states. Similarly, Abdelghany et al. [91] noted that SCOP can generate anxiety, avoidance, and fear and is used to treat nausea and motion sickness in humans. In line with this, the Food and Drug Administration (FDA) lists restlessness, confusion, agitation, and hallucinations as possible side effects of SCOP in humans [92]. Furthermore, Ramandeep et al. [93] described SCOP as a robust and well-defined behavioral model for the study of neurological phenotypes, particularly regarding the generation of anxiety states in zebrafish.

As shown in Figure 6B, chronic administration of OmEO at concentrations of 150 and 300 μL/L improved the latency period of the zebrafish to swim in the top zone of the tank (*p* < 0.05), while at a concentration of 25 μL/L, it showed no effect. Regarding the effects of OmEO on the SCOP-induced amnesic zebrafish model, it was observed that OmEO was able to reduce the latency period only at the highest concentrations (150 and 300 µL/L) (*p* < 0.05). Also, Tukey’s post hoc analyses showed that there is a statistical significance between the experimental groups of fish (III, IV, and V vs. VIII, IX, and X) (*p* < 0.0001), thus highlighting the anxiolytic effects of OmEO on zebrafish. In addition, the anxiolytic effects of OmEO are also supported by data reflecting the amount of time spent in the top zone of the tank. As can be seen in Figure 6C, OmEO at a concentration of 300 μL/L was able to restore the amount of time spent by the fish in the top zone of the tank (*p* < 0.01). However, no significance was observed between the two lower concentrations of OmEO (25 and 150 μL/L) and the SCOP-treated fish in group VI. Simultaneously, treatment with OmEO at a concentration of 300 μL/L, applied to the animals in group X, managed to combat the hypolocomotor effect induced by SCOP by significantly increasing the distance traveled in the top zone of the tank (*p* < 0.05) (Figure 6D), by decreasing the freezing duration (*p* < 0.01) (Figure 6E), and by restoring the average swimming speed of the zebrafish in the NTT (*p* < 0.05) (Figure 6F).

In our study, within the groups treated with GAL, no statistically significant differences were observed compared to the control group or the SCOP treatment group (Figure 6F). This may be attributed to possible endogenous defense mechanisms that mask the differences between groups, such as increases in the activities of antioxidant enzymes or neurotransmitters.

Our results agree with those in the literature. Amaghnouje et al. [94] showed that a hydroethanolic extract of marjoram administered orally to laboratory mice produced significant anxiolytic and antidepressant effects by increasing the exploration of and time spent in the light zone in the light–darkness test, behavior like that induced by bromazepam. In addition, Tripathy et al. [95] have shown that *O. majorana* acts as a tonic agent for the nervous system, helping to strengthen it, and is a plant with anti-anxiety potential that is used in traditional medicine to relieve stress and tension.

### 3.6. Effects of OmEO on Zebrafish’s Spatial Memory, as Assessed in the Y-Maze Test

Spatial memory is the ability to retain information related to significant locations, such as home, food sources, or other relevant places, and is a phenomenon observed in a wide range of species, from invertebrates to humans. This form of memory involves the use of diverse types of information or cues, of which visual landmarks, distances, and directions often play key roles in the storage and retrieval of this spatial information [96]. When it comes to diagnosing AD, it is important to note that spatial disorientation can be one of the first manifestations of this condition. Specifically, more than 60% of patients with this form of dementia have spatial memory deficits and exhibit wandering behaviors [97].

**Figure 7:**
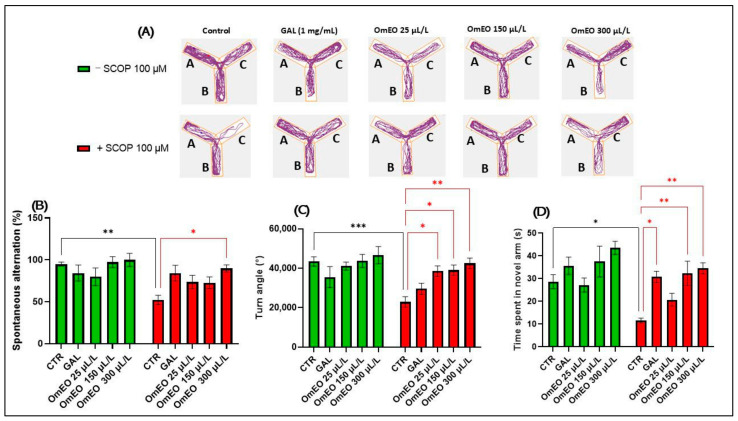
The effects of administering a treatment of *Origanum majorana* essential oil (OmEO), at concentrations of 25, 150, and 300 μL/L, both on naive zebrafish and those treated with scopolamine (SCOP, 100 µM) in the Y-maze. Galantamine (GAL, 1 mg/L) was used as a positive control. (**A**) Graphical representation of the route followed by the zebrafish within the second session of the Y-maze. (**B**) Spontaneous alternation (%). (**C**) Turn angle (°). (**D**) The amount of time spent by the fish in the novel arm (%). Values are expressed as means ± S.E.M., (*n* = 10 animals per group). For Tukey’s post hoc analyses, * *p* < 0.05, ** *p* < 0.01, and *** *p* < 0.001.

To assess the effect of OmEO on spatial memory, the zebrafish were subjected to the Y-maze test, and the spontaneous alternation (%), turn angle (°), and time spent in the novel arm (%) were recorded and analyzed. Figure 7A illustrates specific locomotion patterns observed in the zebrafish in the different groups tested.

In the Y-maze task, Tukey’s post hoc analyses showed that the SCOP-treated zebrafish (groups VI, VII, VIII, IX, and X) showed a lower percentage of spontaneous alternation (*p* < 0.01) (Figure 7B) and a reduced turn angle (°) (*p* < 0.001) (Figure 7C) and spent less time in the novel arm (*p* < 0.05) (Figure 7D) in comparison with the SCOP-free zebrafish (groups I, II, III, IV, and V). These behavioral changes suggest that SCOP affects locomotor activity and induces the impairment of cognitive processes in zebrafish. Previously, numerous studies have highlighted the deficit in memory caused by SCOP in both rodents [98,99] as well as in zebrafish [100]. Tukey’s post hoc analyses revealed no effect of OmEO treatment (groups II, IV, and V) versus the control group (I), suggesting that OmEO cannot affect memory or locomotion in naive fish.

Regarding the impact of OmEO on the fish treated acutely with SCOP (groups VII, IX, and X), it was observed that OmEO only at a concentration of 300 μL/L was able to restore the percentage of spontaneous alternation (*p* < 0.05) (Figure 7B). Also, OmEO had a statistically significant impact on the zebrafish’s locomotion, managing to restore, especially at a 300 μL/L concentration, the turn angle (°) of the zebrafish (*p* < 0.01) but had significant effects also at lower concentrations (25 and 150 μL/L, *p* < 0.05) (Figure 7C). Simultaneously, OmEO, at concentrations of 150 μL/L (*p* < 0.05) and 300 μL/L (*p* < 0.01), was able to completely suppress the effect of the SCOP (*p* < 0.001) on the zebrafish’s memory by increasing the amount of time the fish spent in the novel arm of the Y-maze (Figure 7D). These effects are similar to those of GAL, which significantly increased the percentage of the exploration of the novel arm in the Y-maze (*p* < 0.05) (Figure 7D). These observations suggest the potential of OmEO to prevent memory degradation in zebrafish.

Previous data have indicated that OmEO has a positive impact on memory performance in rats (Aβ1-42) in Y-maze tests [14] and may protect against neuroinflammation-induced cognitive impairment [13].

### 3.7. Effects of OmEO on Zebrafish’s Reference Memory, as Assessed in the Novel Object Recognition Test

Recognition memory is a complex cognitive process essential to the normal functioning of individuals. This aspect of memory allows us to identify objects, events, or people we have previously encountered and distinguish them from new stimuli. However, recognition memory can be impaired in various neurological and psychiatric conditions, such as AD or schizophrenia. Understanding the neurological mechanisms underlying recognition memory is crucial for developing effective therapies. Thus, studies and research continue to explore this area and contribute to progress in the treatment of conditions involving the recognition of memory dysfunctions [101]. Zebrafish have been studied primarily in the recognition of simple geometric shapes, both in 2D and 3D, and reports have shown that they exhibit recognition memory [102].

**Figure 8:**
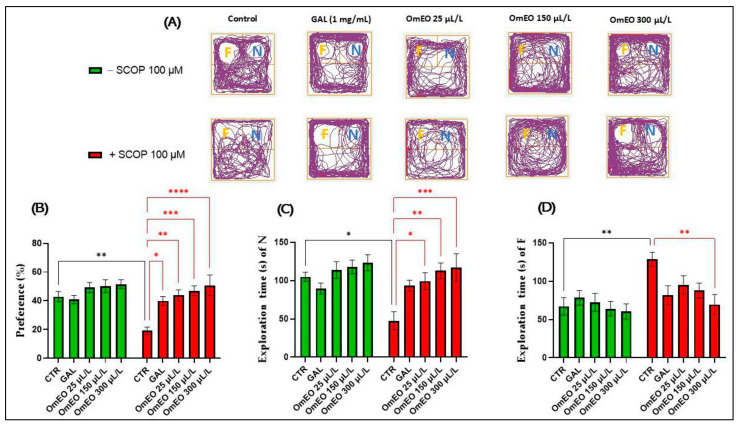
The effects of administering a treatment of *Origanum majorana* essential oil (OmEO), at concentrations of 25, 150, and 300 μL/L, both on native zebrafish and those treated with scopolamine (SCOP, 100 µM) in the novel object recognition test (NOR). Galantamine (GAL, 1 mg/L) was used as a positive control. (**A**) Graphical representation of zebrafish tracks during the test session in the NOR test. The familiar object area is denoted by the initial (F), and the novel object area is denoted by the initial (N). (**B**) Percentage preference (%) for one of the two objects in the NOR test (**C**). Turn angle (°). (**D**) Exploration time (s) of N. Values are expressed as means ± S.E.M., (*n* = 10 animals per group). For Tukey’s post hoc analyses, * *p* < 0.05, ** *p* < 0.01, *** *p* < 0.001, and **** *p* < 0.0001.

In the novel object recognition test (NOR), to assess recognition memory, a fish is subjected to a test in which it is initially exposed to two identical objects. After a delay, the fish is returned to the same environment, which contains one of the original objects and a new object. If the subject spends more time exploring one of these objects, this behavior can be interpreted as a manifestation of memory, as the subject recognizes the object it has seen before [102]. In this research, we aimed to investigate whether OmEO influences reference memory in zebrafish, both wild-type and those acutely treated with SCOP. This assessment involved the use of complex geometric objects, which consisted of yellow and blue cubes. Figure 8A shows the characteristic movement patterns of the zebrafish in the various study groups. The results of Tukey’s post hoc analyses revealed significant decreases in preference percentages (*p* < 0.01) (Figure 8B) and exploration times of object N (*p* < 0.05) (Figure 8C) and a significant increase in the exploration time of F (*p* < 0.01) (Figure 8D) in fish treated with SCOP (100 µM) compared to the control group (according to Figure 8B), indicating the amnesic effect of SCOP on the memory of recognition in zebrafish. Previous data have shown that SCOP affects recognition memory in both monkeys [103] and rodents [104], as well as in laboratory fish [41].

Conversely, the administration of OmEO to zebrafish acutely pretreated with SCOP (groups VIII, IX, and X) significantly increased the percentage of the preference in a dose-dependent manner (*p* < 0.01 at a concentration of 25 µL/L, *p* < 0.001 at concentration of 150 µL/L, and *p* < 0.0001 at a concentration of 300 µL/L) at a level close to that of the GAL treatment (group VII) (*p* < 0.05) (Figure 8B). Also, OmEO was able to increase the N exploration time in the NOR test at all three tested concentrations (*p* < 0.05 for the 25 µL/L concentration, *p* < 0.01 for the 150 µL/L concentration, and *p* < 0.001 for 300 µL/L) (Figure 8C) and to decrease the exploration time of F, especially at the highest concentration (300 µL/L, *p* < 0.01), compared to that of the group treated only with SCOP (VI) (Figure 8D), indicating the amelioration of SCOP-induced negative effects on the fish’s recognition memory. Previously, the beneficial effects of *O. majorana* on recognition memory have also been highlighted in mice [13]. Raafat et al. [105] showed that *O. majorana* extract exhibits protective effects against neurodegeneration by modulating inhibitory glycine receptors (GlyRs) and reversing strychnine toxicity in mice.

### 3.8. Effects of OmEO on Acetylcholinesterase Activity

Cholinergic neurotransmission plays a key role in impairing cognitive function in AD and adult-onset dementia disorders [106]. Numerous studies have identified low levels of acetylcholine in brain regions, such as the cerebral cortex and hippocampus, associated with cognitive processes in animals [107], and AChE activity is increased compared to normal activity levels of this enzyme in the temporal lobe and hippocampus during AD progression [108]. Thus, it is not surprising that the main class of drugs currently used to treat AD (GAL and donepezil) are cholinesterase inhibitors [109]. Therefore, our study wanted to target the impact of OmEO on AChE activity in the brains of both native and SCOP-induced cholinergic-lesion model zebrafish.

**Figure 9:**
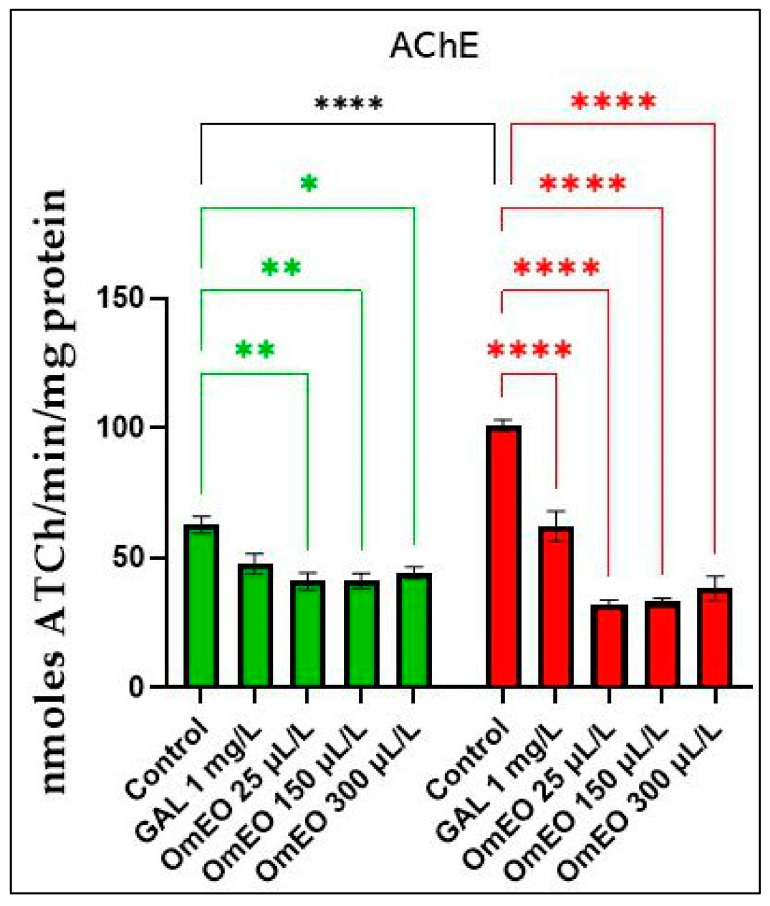
The effects of administering a treatment of *Origanum majorana* essential oil (OmEO), at concentrations of 25, 150, and 300 μL/L, both in native zebrafish and those treated with SCOP (100 µM), on acetylcholinesterase (AChE) specific activities. Galantamine (GAL, 1 mg/L) was used as a reference positive drug. Values represent means ± SEM (*n* = 10). For Tukey’s post hoc analyses, * *p* < 0.05, ** *p* < 0.01 and **** *p* < 0.0001.

As can be seen in Figure 9, compared to the control group (Group I), treatment with SCOP significantly (*p* < 0.0001) increased AChE activity in zebrafish brains in group VI fish. Surprisingly, OmEO administration to naive fish (in groups III, IV, and V) decreased AChE activity compared to that in control fish (*p* < 0.01 at concentrations of 25 µL/L and 150 µL/L and *p* < 0.05 at a concentration of 300 µL/L). This may mean that OmEO can increase the level of acetylcholine in the brain of native zebrafish and, consequently, improve cholinergic neurotransmitter function, suggesting the potential of OmEO in regulating cholinergic neurotransmission. Zebrafish in the groups that first received OmEO at concentrations of 25, 150, and 300 μL/L, followed by subsequent acute treatment with SCOP (100 μM), showed significant reductions, at all three tested concentrations, in AChE activity in the brain (*p* < 0.0001). This observation was compared with that of the group of fish that were treated only with SCOP (group VI). A similar effect was also noted in the group of fish that were treated with SCOP and GAL (group VII). Thus, OmEO can prevent the degradation of the cholinergic neurotransmitter, thus decreasing AChE levels in the brains of amnesic fish and, consequently, can regulate the acetylcholine levels, which are deficient because of SCOP administration.

### 3.9. Effects of OmEO on Oxidative Stress

Oxidative damage is significantly expanded in the brains of AD patients and is correlated with abnormal accumulation of amyloid beta (Aβ) plaques and deposition of neurofibrillary tangles [110]. Under normal conditions, there is a balance between the formation of reactive oxygen species (ROSs) and the antioxidant system. However, this balance is disturbed during pathological scenarios, where antioxidant defense mechanisms become insufficient, leading to the generation of oxidative stress and, often, apoptosis [111]. Oxidative stress is a process that increases in intensity with aging and is caused by an imbalance in the redox balance, involving excessive production of ROSs or dysfunction of the antioxidant system [112]. The mitochondrial electron transport chain consumes almost 98% of the molecular oxygen at the level of the cytochrome oxidase complex. The remaining oxygen is reduced to hydrogen peroxide (H_2_O_2_) and oxygen superoxide radicals (O_2_^−^). During normal metabolism and in various functions, O_2_^−^ and H_2_O_2_ are generated as non-radical oxidants, as well as hypochlorous acid [113]. Mitochondria have regulatory mechanisms to prevent excessive accumulation of ROSs and maintain adequate O_2_^−^ concentrations in intermembrane spaces. These mechanisms include superoxide dismutase (SOD), which acts by the dismutation from O_2_^−^ to H_2_O_2_ and then to water [114]; glutathione peroxidase (GPX), which mostly breaks down O_2_^−^ and converts OH to a less reactive form [36]; and catalase (CAT), which is another important detoxification enzyme found in peroxisomes and helps to remove H_2_O_2_ [115].

**Figure 10:**
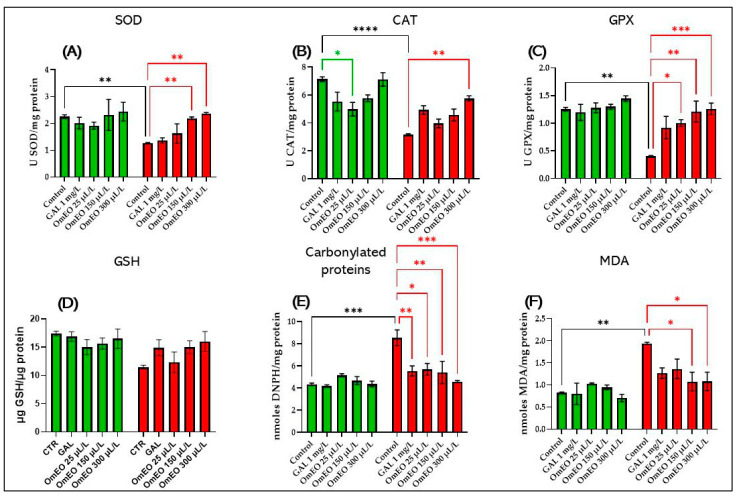
The effects of administering a treatment of *Origanum majorana* essential oil (OmEO), at concentrations of 25, 150, and 300 μL/L, both in native zebrafish and those treated with SCOP (100 µM), on (**A**) superoxide dismutase (SOD), (**B**) catalase (CAT), and (**C**) glutathione peroxidase (GPX) specific activities; (**D**) the level of reduced glutathione (GSH); (**E**) the carbonylated protein level; and (**F**) the malondialdehyde (MDA) content. Galantamine (GAL, 1 mg/L) was used as a positive control. Values represent means ± SEM (*n* = 10). For Tukey’s post hoc analyses, * *p* < 0.05, ** *p* < 0.01, *** *p* < 0.001, and **** *p* < 0.0001.

As can be seen in Figure 10, there was no significant difference between the effects on the native fish in group I and the naive fish treated with OmEO (groups III, IV, and V), in terms of the oxidative status. This may suggest that OmEO does not have adverse effects on the oxidative system in zebrafish. However, Tukey’s post hoc analyses revealed that SCOP (100 µM) treatment for 30 min in zebrafish (group VI) resulted in significant decreases in the specific SOD activity (*p* < 0.01) (Figure 10A), the specific CAT activity (*p* < 0.0001) (Figure 10B), the GPX activity (*p* < 0.01) (Figure 10C), the carbonylated protein content (*p* < 0.001) (Figure 10E), and the malondialdehyde (MDA) level (*p* < 0.01) (Figure 10F) compared to those of the control group (I). However, in our study, administration of SCOP treatment (100 µM) for 30 min did not reveal any statistically significant effect on the reduced glutathione (GSH) content (Figure 10D). Previously, the generation of oxidative stress in the brains of laboratory animals following the administration of SCOP treatments has also been reported in studies involving mice [37], rats [38], and zebrafish [116]. As can be seen in Figure 10, chronic treatment with OmEO showed a protective effect on oxidative stress, by maintaining, under normal physiological conditions, the specific activity of the SOD (*p* < 0.01 at concentrations of 150 and 300 µL/L) (Figure 10A), the CAT activity (*p* < 0.01, 300 µL/L) (Figure 10B), and the GPX activity (*p* < 0.05 at 25 µL/L, *p* < 0.01 at 150 µL/L, and *p* < 0.001 at 300 µL/L) (Figure 10C). OmEO also showed a dose-dependent effect on the content of protein carbonyls (*p* < 0.05 at 25 µL/L, *p* < 0.01 at 150 µL/L, and *p* < 0.001 at 300 µL /L), like that in the GAL treatment (group VII) (*p* < 0.01) (Figure 10E). In addition, OmEO, at concentrations of 150 and 300 μL/L, decreased the level of MDA (*p* < 0.05) (Figure 10F). However, OmEO did not show any effect on the reduced GSH content in the zebrafish brains (Figure 10D). Previous reports have shown that AD individuals have significantly higher MDA levels than age-matched controls [117]. Also, high contents of protein carbonyls in AD-affected individuals have been reported in different brain regions, such as the superior middle temporal gyrus [118], the parietal lobe [119], and the hippocampus [120]. GSH is one of the most significant antioxidant defense molecules in the brain. Both in vitro and animal studies suggest that GSH depletion plays a significant role in oxidative-stress-induced neuronal death and is implicated in neuronal loss in various neurodegenerative diseases, including AD [121].

Typically, GPX activity depends on the availability of GSH to reduce peroxides, and a decrease in GPX activity is associated with reduced GSH levels. However, in our study, no changes in GSH levels were observed, suggesting the existence of more complex regulatory mechanisms of GPX, such as post-translational modifications, including phosphorylation or acetylation, which can influence the enzyme’s efficiency independent of GSH levels. For instance, phosphorylation can activate or inhibit GPX functions, and these changes may be influenced by intracellular signaling in response to oxidative stress or environmental factors. Although GSH is the primary substrate for GPX, it can also utilize other compounds for peroxide reduction, such as lipid peroxides or H_2_O_2_. In a redox-imbalanced environment, changes in the concentrations of these substrates could influence GPX activity, even in the absence of significant changes in GSH levels [122]. Additionally, GPX activity can be regulated within the antioxidant network, which includes enzymes, such as SOD and CAT. Modifications in the activities of these enzymes can indirectly affect GPX activity, even when GSH levels remain constant. For example, in cases of imbalance between SOD and CAT, GPX may be more heavily recruited to counteract oxidative stress, leading to changes in its activity [123,124]. These mechanisms suggest that the regulation of GPX activity is a complex process, influenced by multiple pathways and factors, and not solely dependent on GSH levels.

Treatment with GAL was found to be effective only in modulating the level of protein carbonylation (*p* < 0.001) (Figure 10E), indirectly suggesting its antioxidant properties.

The antioxidant effects of *O. majorana* L have also been previously mentioned in Taha’s study [125], where pretreatment with *O. majorana* L. extract significantly ameliorated acetic acid-induced ulcerative colitis. This effect could be attributed to the protective, antioxidant, anti-inflammatory, and anti-apoptotic properties of the extract. Also, high contents of protein carbonyls in AD-affected individuals have been reported in different brain regions, such as the superior middle temporal gyrus, and *O. majorana* L. and sulfasalazine had protective effects against oxidative stress in ulcerative colitis induced by acetic acid in rats, manifested by decreases in MDA levels and increases in the activities of some antioxidant enzymes (SOD, CAT, and GSH). These effects were mediated through the Nrf2/HO-1 signaling pathway and by re-inducing inflammation in the colon, inhibiting important markers of inflammation, such as NFκB, TNFα, IL-1β, IL6, IL23, IL17, COX-2, and iNOS, which resulted in a downregulation of the JAK2/STAT3 signaling pathway. Also, the authors of that study reported a decrease in the number of epithelial cells that entered the process of apoptosis through caspase-3. The antioxidant effects of *O. majorana* L. were also mentioned by Seif et al. [126] and Wagdy et al. [13].

The variability of oxidative stress markers observed in this study reflects the complexity of redox processes and the methodological limitations associated with them. First, individual markers, such as SOD, CAT, GPX, MDA, and carbonylated proteins, responded differently to treatments, which can be explained by compensatory mechanisms of the antioxidant system, differential sensitivity of the markers, and possible post-translational modifications, such as GPX phosphorylation [123,127]. The lack of significant effects on GSH levels, an essential substrate for GPX, suggests the presence of more complex regulatory mechanisms, including the use of other substrates (e.g., lipid peroxides) or interactions between antioxidant enzymes [128]. Additionally, variability between individuals can be attributed to genetic, physiological, or environmental differences, while the duration of the treatment and the sensitivity of the biochemical methods used may influence the accuracy of the results. Furthermore, the antioxidant effects of OmEO and GAL, although evident for certain markers, were not uniform, indicating potential mechanistic differences between the two treatments, including activation of the Nrf2/HO-1 pathway for OmEO, and the specific antioxidant properties of GAL [129].

### 3.10. Correlation Analysis Between Behavioral and Biochemical Parameters

Pearson’s correlation coefficient (r) was used to investigate the relationships between behavioral scores and enzymatic activities and lipid peroxidation, which included variables such as the amount of time spent in the top zone of the tank in the NTT (s), the amount of time spent in the novel arm of the Y-maze (s), preference (%) in the NOR, AChE activity, SOD specific activity, CAT specific activity, GPX specific activity, and the level of carbonylated proteins in the fish brain relative to the level of MDA.

**Figure 11:**
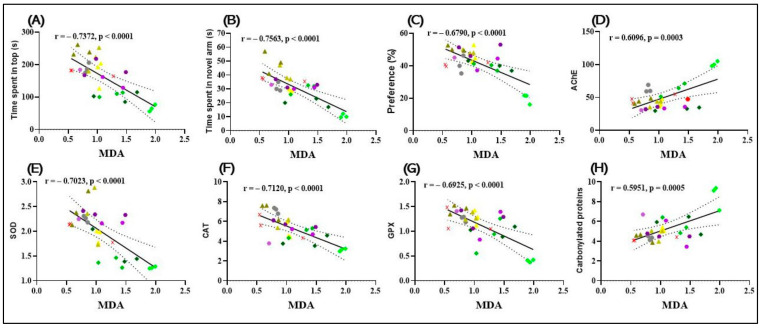
Correlation analysis between behavioral and biochemical parameters (Pearson’s correlation). Data shown are (**A**) amount of time spent by fish in the top zone of the NTT vs. MDA (*n* = 10, r = −0.7372, *p* < 0.0001); (**B**) amount of time spent by fish in the novel arm of the Y-maze vs. MDA (*n* = 10, r = −0,7563, *p* < 0.0001); (**C**) preference (%) in the NOR vs. MDA (*n* = 10, r = −0.6790, *p* < 0.0001); (**D**) AChE vs. MDA (*n* = 10, r = 0.6096, *p* < 0.001); (**E**) SOD vs. MDA (*n*= 10, r = −0.7023, *p* < 0.0001); (**F**) CAT vs. MDA ( *n* = 10, r = −0.7120, *p <* 0.0001); (**G**) GPX vs. MDA (*n* = 10, r = −0.6925, *p* < 0.0001); and (**H**) carbonylated proteins vs. MDA (*n* = 10, r = 0.5951, *p* < 0.001) (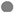) Control, (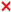) Galantamine (GAL, 1 mg/mL), (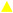) *Origanum majorana* essential oil (OmEO, 1 μL/L), (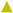) OmEO 3 μL/L, (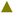) OmEO 6 μL/L, (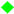) Scopolamine (SCOP, 100 μM), (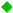) SCOP (100 μM) + GAL 1 mg/mL, (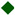) SCOP (100 μM) + OmEO 1 μL/L, (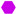) SCOP (100 μM) + OmEO 3 μL/L, (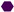) SCOP (100 μM) + OmEO 6 μL/L.

The results showed a significant negative correlation between the amount of time spent in the top zone in the NTT (Figure 11A), the amount of time spent exploring the novel arm in the Y-maze (Figure 11B), the preference (%) in the NOR (Figure 11C), SOD specific activity (Figure 11E), CAT specific activity (Figure 11F), and GPX specific activity (Figure 11G) relative to the MDA level, with correlation coefficients (r) ranging between −0.6790 for GPX vs. MDA and −0.7563 between the amount of time spent exploring the novel arm in the Y-maze and the level of MDA.

In contrast, the specific activity of the AChE (Figure 11D) and the level of carbonylated proteins (Figure 11H) showed a significantly positive correlation with the level of MDA, with correlation coefficients (r) between 0.5951 and 0.6096. These results suggest a complex relationship between behavior, enzymatic activities, and lipid peroxidation that may provide important insights into pathophysiological mechanisms involved in treatment responses and effects on the nervous system. Thus, because of its impact on markers of oxidative stress and improvements observed in behavioral tests, OmEO could represent a promising option for the treatment of symptoms related to amnesia and anxiety.

## 4. Discussion

In this study, the chemical composition of OmEO was analyzed using GC/MS, identifying 17 compounds that accounted for 93.52% of the oil. Among the major compounds were trans-sabinene-hydrate (36.11%), terpinen-4-ol (17.97%), linalyl acetate (9.18%), caryophyllene oxide (8.25%), and α-terpineol (6.17%). These substances are associated with antioxidant effects and cognitive function enhancement. The ADMET (absorption, distribution, metabolism, excretion, and toxicity) properties of certain chemical compounds, including SCOP, GAL, and major components of OmEO (trans-sabinene-hydrate, terpinen-4-ol, linalyl acetate, caryophyllene oxide, and α-terpineol), were assessed using computational platforms to predict the behaviors of these compounds in the human body. The results showed that GAL and OmEO compounds had high absorption and blood–brain barrier permeability, while SCOP exhibited low permeability. Additionally, GAL and SCOP interact with the CYP3A4 substrate, while caryophyllene oxide interacts with CYP1A2. For example, terpinene-4-ol and α-terpineol were identified as having low toxicities but potential carcinogenicities, while SCOP and GAL presented higher toxicities at the hepatic and respiratory levels. Toxicity predictions indicated that α-terpineol and terpinen-4-ol had higher tolerances compared to those of SCOP and GAL, which displayed lower tolerance. Toxicity pathway analysis revealed various interactions of the compounds with nuclear receptors and stress response pathways, as exemplified by how caryophyllene oxide interacts with nuclear receptors and influences oxidative stress mechanisms.

In our study, we used zebrafish as a model organism, which represents a significant limitation as results obtained from animal models are not always directly translatable to humans. Although zebrafish provide valuable experimental models because of their biological characteristics, such as transparency, rapid growth, and genetic simplicity, there are significant differences between their physiology and that of mammals, including humans. These differences can affect how they respond to treatments, and the observed effects in this model cannot always be directly extrapolated to higher species. Furthermore, mammalian studies are essential for validating these results and gaining a deeper understanding of the mechanisms by which *O. majorana* and its active compounds may influence cognitive functions and oxidative stress in the context of human pathologies, particularly AD. Another significant limitation of our study is the short duration of the experiment, which may not be sufficient to evaluate the long-term effects of OmEO administration. Given the progressive nature of neurodegenerative diseases, like AD, which develop over several years, longitudinal studies are necessary to understand better the long-term impacts of the treatment on the cognitive state and oxidative processes. OmEO administration improved mental performance in naive zebrafish and those subjected to SCOP-induced amnesia, as demonstrated by behavioral tests (Y-maze and NOR). Previous research has demonstrated that OmEO exerts a beneficial effect on memory performance in rats subjected to Aβ1-42-induced cognitive deficits [14]. The essential oil also showed potential in reducing SCOP-induced anxiety, as evidenced by increased time spent in the upper zone of the NTT. Tripathy et al. [95] previously highlighted the anxiogenic effects of OmEO. Additionally, our results indicated that OmEO may protect zebrafish brains from the effects of oxidative stress by modulating SOD, CAT, and GPX activities, as well as reducing protein carbonyl levels and MDA. The antioxidant properties of *O. majorana* L. have also been highlighted by Seif et al. [126] and Wagdy et al. [13]. Regarding the translational implications of using OmEO for AD-related treatments, the obtained data suggest the significant therapeutic potential of OmEO in ameliorating cognitive dysfunctions and oxidative stress associated with this condition. The inhibition of AChE activity by OmEO may contribute to increased ACh levels, a neurotransmitter involved in learning and memory processes, which are significantly affected in AD. Reducing oxidative stress by decreasing markers of lipid peroxidation and carbonylated proteins supports the hypothesis that OmEO could offer neuronal protection by inhibiting oxidative damage, which plays a central role in disease progression. However, to support its use as a treatment in AD, further studies are needed to investigate the biodisponibility, pharmacokinetics, and long-term efficacy of OmEO, as well as its interactions with other pharmaceutical therapies used for neurodegenerative diseases. Regarding safety, the administration of OmEO may present certain risks that must be considered before recommending it for therapeutic use. Although in our study, OmEO showed beneficial effects in improving cognitive performance and reducing oxidative stress, essential oils can cause adverse reactions, including skin irritation or allergic reactions, especially at high doses or with prolonged administration. Interactions with other medications may also occur, highlighting the need for thorough safety assessments before clinical implementation. High-dose or long-term administration may have toxic effects, and these risks must be investigated through toxicity and safety studies to ensure safe use in patients, particularly those with comorbidities or those undergoing specific pharmaceutical treatments. Thus, although our study suggests the promising potentials of *O. majorana* and its essential oil in AD treatments, further research is needed, including preclinical mammalian studies, long-term safety assessments, and clinical investigations, to confirm their efficacy and safety in neurodegenerative treatments.

## 5. Conclusions

This study predicted the pharmacokinetic properties of the main compounds (trans-sabinene hydrate, terpinen-4-ol, linalyl acetate, caryophyllene oxide, and α-terpineol) in OmEO, using in silico techniques. Moreover, this study aimed to investigate the effects of OmEO on memory processes in the animal model of dementia, as exemplified by the zebrafish. The results confirmed the neuroprotective potentials of the main compounds in OmEO, and some of the identified compounds showed a strong binding affinity with target proteins compared to the reference drug (GAL). Also, the ADMET results showed that all five compounds in OmEO show high absorption and distribution and can cross the BBB easily. Also, the toxicity data indicated that none of the compounds shows hepatotoxicity, but terpinen-4-ol and α-terpineol could show carcinogenicity, and linalyl acetate could negatively affect the respiratory system.

In contrast to our in vivo test results, the results of our research indicated no effects on the behavior and antioxidant status of naive zebrafish after chronic treatment with OmEO at concentrations of 25, 150, and 300 μL/L. However, in the zebrafish model of amnesia induced by immersion in SCOP (100 μM), OmEO, especially at higher concentrations, namely, 300 μL/L, had a significant behavioral ameliorating effect on anxious zebrafish in the NTT test by restoring the top zone exploration time and reducing freezing periods. OmEO treatment also restored spontaneous alternation (%) and significantly prolonged the exploration time of the novel arm in Y-maze tasks, as well as the zebrafish’s preference in the NOR. At the same time, OmEO treatment effectively restored the antioxidant defense mechanism by increasing the brain’s antioxidant activity levels (SOD, CAT, and GPX) and GSH content and reducing carbonylated protein levels and the MDA content. OmEO also inhibited the activity of AChE, which led to an improvement in cognitive activity. Therefore, our study brought forth, for the first time, the anti-amnesic and neuroprotective effects of OmEO on SCOP-treated zebrafish and presented OmEO as a potential natural candidate for the prevention or control of anxiety-like behavior and amnesia states. In addition, these results pave the way for further research toward the development of natural treatments for cognitive and behavioral disorders.

Future research directions should include the validation of these effects in vivo using rodent models, clinical studies to assess efficacy in humans, the exploration of interactions with other treatments, and a deeper investigation into the molecular mechanisms involved. These studies will contribute to the development of innovative therapies for cognitive disorders.

## Figures and Tables

**Table 1:** Identification of OmEO compounds by GC/MS.

No.	Retention Time (min)	Compound Name	Molecular Formula	RI_Cal_	RI_Lit_	Composition (%)	Method of Identification
1	8.530	camphene	C_10_H_16_	959	953	0.65	RI, MS
2	10.114	*p*-cymene	C_10_H_14_	1015	1018	3.18	RI, MS
3	11.452	γ-terpinene	C_10_H_16_	1058	1059	3.51	RI, MS
4	12.506	trans-sabinene-hydrate	C_10_H_18_O	1092	1098	36.11	RI, MS
5	13.137	dehydro sabinene ketone	C_9_H_12_O	1112	1117	1.12	RI, MS
6	13.703	3-isothujenol	C_10_H_18_O	1130	1134	0.82	RI, MS
7	14.914	terpinen-4-ol	C_10_H_18_O	1169	1174	17.97	RI, MS
8	15.335	α-terpineol	C_10_H_18_O	1183	1186	6.17	RI, MS
9	17.163	linalyl acetate	C_12_H_20_O_2_	1245	1254	9.18	RI, MS
10	17.659	u.i.	-	1263	-	2.55	-
11	18.199	bornyl acetate	C_15_H_18_O_2_	1281	1280	1.24	RI, MS
12	18.485	3-thujanyl-acetate	C_12_H_20_O_2_	1291	1295	0.53	RI, MS
13	18.560	u.i.	-	1294	-	2.10	-
14	20.200	u.i.	-	1351	-	1.94	-
15	23.502	u.i.	-	1475	-	2.45	-
16	25.961	spathulenol	C_15_H_24_O	1571	1577	2.24	RI, MS
17	26.103	caryophyllene oxide	C_15_H_24_O	1576	1581	8.25	RI, MS

Cal: calculated; Lit: literature; u.i.: unidentified; RI: retention index; MS: mass spectrum.

**Table 2:** Predictions of Caco2 permeability, intestinal absorption (human), skin permeability, VDss (human), BBB permeability, CNS permeability, CYP3A4 substrate, CYP1A2 inhibition, total clearance, renal OCT2 substrate, maximum tolerated dose (human), oral rat acute toxicity (LD50), oral rat chronic toxicity (LOAEL), hepatotoxicity, skin sensitization, carcinogenicity, and respiratory toxicity.

Property	Compound Model Name	SCOP	GAL	Trans-Sabinene Hydrate	Terpinen-4-ol	Linalyl Acetate	Caryophyllene Oxide	α-Terpineol	Unit
Absorption	Caco2 permeability	0.059	1.594	1.494	1.502	1.635	1.414	1.489	Numeric (log Papp in 10^−6^ cm/s)
	Intestinal absorption (human)	72.626	94.994	94.786	94.014	95.275	95.669	94.183	Numeric (% Absorbed)
	Skin permeability	−4.097	−3.75	−2.128	−2.182	−1.968	−3.061	−2.418	Numeric (log Kp)
Distribution	VDss (human)	0.583	0.89	0.351	0.21	0.069	0.564	0.207	Numeric (log L/kg)
	Fraction unbound (human)	0.414	0.36	0.469	0.514	0.423	0.327	0.565	Numeric (Fu)
	BBB permeability	−0.043	−0.081	0.663	0.563	0.516	0.647	0.305	Numeric (log BB)
	CNS permeability	−3.031	−2.511	−2.24	−2.473	−2.379	−2.521	−2.807	Numeric (log PS)
Metabolism	CYP3A4 substrate	Yes	Yes	No	No	No	No	No	Categorical (Yes/No)
	CYP1A2 inhibitor	No	No	No	No	No	Yes	No	Categorical (Yes/No)
Excretion	Total clearance	1.096	0.991	1.011	1.269	1.627	0.905	1.219	Numeric (log ml/min/kg)
	Renal OCT2 substrate	No	Yes	No	No	No	No	No	Categorical (Yes/No)
Toxicity	Maximum tolerated dose (human)	−0.319	−0.423	0.637	0.857	0.547	0.148	0.886	Numeric (log mg/kg/day)
	Oral rat acute toxicity (LD50)	2.234	2.728	1.703	1.811	1.729	1.548	1.923	Numeric (mol/kg)
	Oral Rat Chronic toxicity (LOAEL)	0.736	0.966	1.926	2.02	2.256	1.224	1.945	Numeric (log mg/kg_bw/day)
	Hepatotoxicity	No	Yes	No	No	No	No	No	Categorical (Yes/No)
	Skin sensitization	No	No	Yes	Yes	Yes	Yes	Yes	Categorical (Yes/No)
	Carcinogenicity	No	No	No	Yes/No	No	No	Yes	Categorical (Yes/No)
	Respiratory toxicity	Yes/No	Yes	No	No	Yes	No	No	Categorical (Yes/No)

**Table 3:** Prediction of Tox21 pathway.

Compound Tox21 Pathway	SCOP	GAL	Trans-Sabinene Hydrate	Terpinen-4-ol	Linalyl Acetate	Caryophyllene Oxide	α-Terpineol
NR-AR	No	Yes/No	No	No	No	No	No
NR-AR-LBD	No	No	No	No	No	No	No
NR-AhR	No	Yes	No	No	No	No	No
NR-ER	Yes/No	No	No	No	No	No	No
NR-ER-LBD	Yes	No	No	No	Yes/No	No	No
Surface Area	No	No	No	No	No	No	No
NR-PPAR-gamma	No	No	No	No	No	No	No
SR-ARE	No	No	No	No	No	No	No
SR-ATAD5	No	No	No	No	No	No	No
SR-HSE	No	No	No	No	No	Yes/No	No
MMP	No	No	No	No	No	No	No
SR-p53	No	No	No	No	No	No	No

NR-AR—androgen receptor; NR-AR-LBD—androgen receptor ligand-binding domain; NR-AhR—aryl hydrocarbon receptor; NR-ER—estrogen domain; NR-ER-LBD—estrogen domain ligand-binding domain; NR-PPAR-gamma—peroxisome proliferator-activated receptor gamma; SR-ARE—antioxidant response element; SR-ATAD5—ATPase family AAA domain containing 5; SR-HSE—heat shock factor response element; MMP—mitochondrial membrane potential; SR-p53—tumor protein P53.

**Table 4:** Compounds’ properties.

Descriptor	SCOP	GAL	Trans-Sabinene Hydrate	Terpinen-4-ol	Linalyl Acetate	Caryophyllene Oxide	α-Terpineol
Molecular Weight	303.358	287.359	154.253	154.253	196.29	220.356	154.253
LogP	0.9181	1.8503	2.1935	2.5037	3.2406	3.9364	2.5037
Rotatable Bonds	4	1	1	1	5	0	1
Acceptors	5	4	1	1	2	1	1
Donors	1	1	1	1	0	0	1
Surface Area	129.371	1.8503	68.806	69.123	86.649	99.255	69.123

**Table 5:** Estimated pharmacokinetic parameters of drug-likeness and medicinal chemistry properties of the compounds analyzed in this study.

Drug Likeness	SCOP	GAL	Trans-Sabinene Hydrate	Terpinen-4-ol	Linalyl Acetate	Caryophyllene Oxide	α-Terpineol
Lipinski	Yes; 0 violations	Yes; 0 violations	Yes; 0 violations	Yes; 0 violations	Yes; 0 violations	Yes; 0 violations	Yes; 0 violations
Ghose	Yes	Yes	No; 1 violation: MW < 160	No; 1 violation: MW < 160	Yes	Yes	No; 1 violation: MW < 160
Veber	Yes	Yes	Yes	Yes	Yes	Yes	Yes Yes
Egan	Yes	Yes	Yes	Yes	Yes	Yes	No; 2 violations: MW < 200, Heteroatoms < 2
Muegge	Yes	Yes	No; 2 violations: MW < 200, Heteroatoms < 2	No; 2 violations: MW < 200, Heteroatoms < 2	No; 1 violation: Heteroatoms < 2	No; 1 violation: MW < 200	
Bioavailability Score	0.55	0.55	0.55	0.55	0.55	0.55	0.55

## Data Availability

The data are contained within this article.
